# Stress response to handling is short lived but may reflect personalities in a wild, Critically Endangered tortoise species

**DOI:** 10.1093/conphys/cox008

**Published:** 2017-03-04

**Authors:** Andrea F. T. Currylow, Edward E. Louis, Daniel E. Crocker

**Affiliations:** 1 ACEcological Research and Consulting, Oceanside, CA, USA; 2 Madagascar Biodiversity Partnership, Conservation Genetics Department, Omaha's Henry Doorly Zoo and Aquarium, Omaha, NE, USA; 3 Department of Biology, Sonoma State University, Rohnert Park, CA, USA

**Keywords:** Animal personality, *Astrochelys*, corticosterone, ecophysiology, Madagascar, radiated tortoise

## Abstract

We investigated the acute stress response associated with animal personalities by measuring plasma glucocorticoids throughout handling and collected ~2 years of movement and behavioural data in a wild, Critically Endangered animal, *Astrochelys radiata* (radiated tortoise). To determine whether our standard, brief conscientious handling procedures induce a stress response in our target species, we applied a stressor by way of initial animal processing and deployment of telemetry equipment. During surveys and processing, we sampled animals immediately upon detection, again after completing transmitter attachment and processing, and a final time the following day. We then used radiotelemetry to follow a subset of the animals for 22 months while collecting behavioural, climatic and location data. We found that brief and conscientious handling did not illicit consistent changes in plasma concentrations of the stress hormone corticosterone (CORT) but did reveal tremendous individual variation in response. The CORT concentration ranged more than 200-fold after imposing the stressor and returned to near-baseline values by the following day. When we accounted for the wide variation by calculating the degree of each individual's stress response relative to its baseline over its processing time, we discovered two non-overlapping physiological response types; those in which CORT concentrations increased dramatically in response to handling (219 ± 89.8 pg/ml/min) and those in which CORT varied only slightly (5.3 ± 8.9 pg/ml/min). The response types (strong vs. mild) also predicted body condition, home range size, activity, and behavioural tendencies. The degree of the individual's stress response in this species may be one component of correlated physiological and behavioural traits (animal personalities), which have previously been obscured in other chelonian studies by the use of mean values and should be considered in future conservation management applications for chelonian species.

## Teaser

We detected a short-term stress response to our handling procedures in only half of wild, critically endangered radiated tortoises. After collecting nearly 2 years of monitoring data, we found that those initial stress responses were also reflective of differences in traits, i.e. body sizes, behaviours, home ranges and movements.

## Introduction

Studying wild animals in their natural habitats poses challenges to researchers. Among those challenges is the design of wildlife ecology studies that curtail any potential influence the research procedures themselves may have on the variables of interest. Handling naive animals for equipment attachment or data collection will probably induce physiological stress in the animal, which may carry unknown longer-lasting effects. When an animal initially perceives a stressor, the hypothalamic–pituitary–adrenal axis is activated, and within minutes, increased concentrations of steroid hormones can be detected in the bloodstream. If the perceived stressor persists, there is an associated increase in circulating glucocorticoid concentrations, which triggers a cascade of changes in the animal, such as altering reproduction and immune function (Wingfield *et al*., 1998; Sapolsky *et al*., 2000). Yet even conspecifics within a single population can vary widely from ‘the golden mean’ (Williams, 2008), and the stress response can vary in magnitude and persistence depending on the individual animal (Cockrem and Silverin, 2002; Cockrem, 2007, 2013; Williams, 2008). It is therefore important to follow individuals over time and attempt to gain an understanding of typical behaviours compared with the degree and breadth of responses to a particular stressor.

Endocrine regulation is the proximate mechanism of the stress response but can reflect the ultimate, evolutionary mechanisms, in which entire suites of behavioural traits may have evolved in unison to increase fitness in response to environmental factors (Zera *et al*., 2007). How an animal responds to a short-term stressor may reflect its longer-term ability to acclimate to a changing environment in the face of disease, human encroachment, and dwindling resources. In systems that involve species of special conservation concern, individual behavioural traits may be the crucial component driving species survival, yet they have not been studied specifically in most systems.

Grouping of conspecific trait variation is known by various terms (e.g. animal personalities, temperaments, behavioural syndromes or coping styles; Koolhaas *et al*., 1999; Dall *et al*., 2004; Sih *et al*., 2004; Réale *et al*., 2007). The traits of interest are characterized as follows: (i) distinct, associated behaviours that are consistent over time and in differing situations; (i) composed of multiple correlated physiological, behavioural and/or life-history traits; and (iii) objectively measureable (Sih *et al*., 2004; Groothuis and Carere, 2005). These groupings (i.e. personalities) help researchers to understand complex and differing responses of individuals to stochastic environmental or anthropogenic events, are likely to be adaptive (have direct effects on individual fitness; e.g. Boon *et al*., 2007), and may lead to speciation (Wolf *et al*., 2007; Biro and Stamps, 2008; Ingley and Johnson, 2014; Lapiedra *et al*., 2017; but see Crews, 2013).

To investigate the efficacy of conscientious handling procedures and individual responses to those procedures in a species of high conservation concern, we studied the radiated tortoise (*Astrochelys radiata*), an animal designated as Critically Endangered by the International Union for the Conservation of Nature (IUCN) and endemic to the southwestern coastline of Madagascar (Leuteritz and Paquette, 2013; IUCN, 2016). The species has recently experienced range-wide population declines (O'Brien *et al*., 2003; Rafeliarisoa *et al*., 2013; Walker *et al*., 2014), yet little ecological or behavioural work has been done (Leuteritz and Ravolanaivo, 2005; Leuteritz and Paquette, 2008), and no physiological studies have been undertaken in natural conditions. The species’ current and dramatic declines have sparked conservation interest for the animal's wild ecology and physiology, and several studies along with conservation efforts have been initiated (Currylow *et al*., 2014; Randriamahazo *et al*., 2014; Currylow and Stanford, 2015).

Given that studies can themselves cause stress-related impacts, a basic understanding of the study species’ naturally existing physiological state (baseline) should be the first step for these research studies, followed by longer-term monitoring. The aims of the present study were as follows: (i) to determine baseline concentrations of the primary stress glucocorticoid, corticosterone (CORT), in wild, free-ranging tortoises within relatively undisturbed habitat; (ii) to investigate the timing and degree of the stress response in wild tortoises attributable to typical handling procedures during equipment deployment; and (iii) to identify longer-term behaviour patterns that may be correlated with individual animal personalities as evident in differences in stress responses.

## Materials and methods

### Study system

We conducted a field study on wild, free-ranging radiated tortoises located near the community of Lavavolo in southern Madagascar from February 2012 to November 2013. The area is classified as Spiny Forest and is characterized by the dominant plant families of Euphorbiaceae and Didiereaceae (Fenn, 2003), but is modified by pervasive patches of invasive *Opuntia* spp. cactus. The area is divided into three habitat type zones (dunes, littoral, and plateau), with varying levels of impact from *Opuntia*. The dune zone is most heavily impacted by human use, followed by the littoral zone, and finally, the more pristine plateau. Radiated tortoises are crepuscular, spending much of the hottest daytime hours in the shade of brush and trees. They typically become more active, feeding and breeding, in the rainy season from January to April, and reduce activities for the remainder of the year, during the dry season.

### Sampling and data collection

To investigate the physiological stress response to routine but conscientious handling and processing procedures, we sampled animals at three time points during animal processing, as follows: (i) immediately upon location of the animal and generally within 4 min of detection (baseline); (ii) after processing, immediately before release of the animal to its capture location (post-handling); and (iii) immediately upon relocation ~24 h after initial processing (post-24 h). Blood samples were collected using a 2.5 cm, 22-gauge needle on a 3 ml syringe via the subcarapacial sinus (Hernandez-Divers *et al*., 2002). The time to blood sample from first disturbance (upon first discovering the animal during survey before setting up a ‘processing station’ nearby) was noted upon each sampling event using a stopwatch. Stress to the animals was imposed through standard field processing, including manipulation of the animal's orientation, brief physical restraint of the legs, blood collection, and immobilization using a pedestal during collection of morphometric data and transmitter deployment. During processing, we epoxied transmitters (model RI-2C2, Holohil Systems, Ltd, Carp, ON, Canada; affixed using J-B Kwik two-part epoxy, J-B Weld, Inc.) to the fifth costal scute and recorded straight carapace length (SCL; measured in millimetres at the mid-line from nuchal fork to pygle using graduated aluminum tree callipers, Haglöf, Sweden), weight (in kilograms using Pesola^®^ spring scales) and GPS location (using a hand-held Garmin Ltd Rhino^®^ GPS unit). Other data collected included demographics, weather, habitat characteristics, and observed animal behaviour before disturbance (following Currylow *et al*., 2015). We aimed to keep the handling time to within 20 min, then resampled the next day for the post-24 h sampling event. To standardize weights and sizes across the study animals, we calculated body condition scores (BCS) for each animal by dividing the animal's weight by its carapacial length. Generally, the higher the BCS, the healthier the animal (heftier weights for body size).

We trained two local villagers to use radiotelemetry and regularly track (usually one to three times per week) a subset of the sampled animals for 22 months after handling. Each time an animal was tracked, trackers collected temperature (air and ground), humidity (ground), vegetative cover, GPS location and observed animal behaviour (basking, eating, resting in the open, resting in shrub, walking, or other). Trackers were careful not to touch or otherwise disturb the animals during radio-relocation in order to avoid influencing behaviours.

Tracking relocation data were analysed in ArcGIS 10.2.2 for Desktop (version 10.2.2.3552 © 2014 Esri Inc., Redlands, CA, USA) using the Direction Distribution function in Spatial Statistics for creating core Standard Deviational Ellipses for each animal. The areas of these core home range ellipses encompass 95% (95% kernel density ellipse) of all the locations for each animal. Kernel density ellipses are widely used to represent home range sizes in the literature, and we used them here for comparison purposes. To assess ranging activity, we used ArcGIS to calculate the steplengths (the distances between relocation points) for each animal. The use of steplengths allows researchers to quantify the frequency and extent of regular movements.

### Sample processing and corticosterone enzyme immunosorbent assays

We collected blood samples from the subcarapacial sinus of free-ranging male, female, and sub-adult radiated tortoises in February 2012. We stored the samples in heparinized vacutainers inside a cooler bag until further processing at camp (usually ≤5 h). Samples were centrifuged and plasma was transferred to cryovials for immediate storage in liquid nitrogen. All samples were kept in liquid nitrogen until they could be transferred to a −20°C freezer ~3 weeks later. Samples were transported on dry ice from Madagascar to the US and stored at −80°C until analysis ~3 months later.

The primary stress glucocorticoid, commonly known as the stress hormone, in herpetofauna and birds is CORT, whereas it is cortisol in most mammals and fish. We assayed plasma CORT concentrations using enzyme immunoassay (EIA) kits (Cayman Chemical #500655, Ann Arbor, MI, USA). Each sample was analysed in duplicate or in triplicate, until the coefficient of variation for each sample was ≤15%, with an average intra-assay coefficient of variation ≤5%. The assay platform antibody was validated for use in radiated tortoises. Serial dilutions of pooled samples exhibited displacement parallel to that of the standard curve. Pooled samples spiked with standards demonstrated excellent accuracy and recovery (*r*^2^ = 0.99, mean recovery = 97%).

### Statistical analyses

To test whether we elicited a stress response in the study animals attributable to handling and equipment attachment, we modelled CORT concentration changes with sample timing using a linear mixed model with animal identity as a random effect. We tested whether plasma baseline CORT concentrations varied according to circadian variation, handling time before sample collection, home range size, sex, body condition, or age class. We used linear mixed models, with CORT concentration as the dependent variable and time of day, time to blood, sex, weight, SCL, BCS, and age class as the predictors, again with animal identity as the random variable to account for repeated measures. We assessed individual variation of CORT concentrations during handling and compared stress responses between individuals by calculating the degree of the stress response (percentage change relative to the baseline sample) and the slope or rate of change in CORT [(post-handling CORT – baseline CORT)/time to blood sample] for each individual. We used those slopes to identify the different response types and retested the dependent variables (sample period, sample timing, home range size, sex, BCS, and age class). To determine whether observations of animal movements or behaviours were distinguishable between the groups, we logarithmically transformed data for normality and used a χ^2^ contingency analysis followed by a correspondence analysis to assess most closely associated dependent and predictor variables. Ideally, we would explore factors that describe correlation between behavioural traits; however, given sample size limitations relative to the number of trait variables, we compared mean values across variables among the two groups defined by the magnitude of the CORT response. All statistical analyses were run using JMP Pro 12.0.1 (© 2015, SAS Institute Inc.). Significance was determined at *P* ≤ 0.05.

## Results

We collected 44 plasma samples from 17 individual radiated tortoises (seven males, eight females, and two sub-adults). We aimed to collect three samples (baseline, post-handling, and post-24 h) from each individual; however, the extreme and isolated field conditions (both in physically sampling animals and rigorous sample processing and storage) did not permit perfect sampling. Two animals were not sampled post-handling, three were not sampled post-24 h, and two sample tubes failed during storage and transport. Initial samples were collected within 3.3 ± 1.0 min (which includes the time between discovering the animal and setting up a processing station nearby before handling), post-handling sample timing averaged 17.1 ± 2.3 min, and the post-24 h samples were collected on average within 2.1 ± 0.5 min of relocation of the animal.

### Corticosterone concentrations

**Figure 1:**
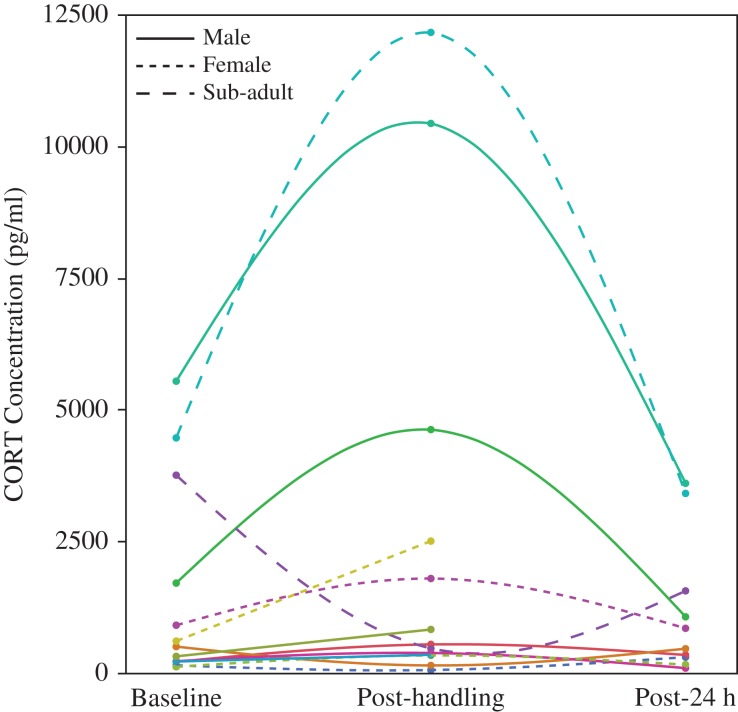
Corticosterone (CORT) concentration measured for each individual *Astrochelys radiata* at each sample time in February, 2012. Trend lines connect individuals’ samples.

**Figure 2:**
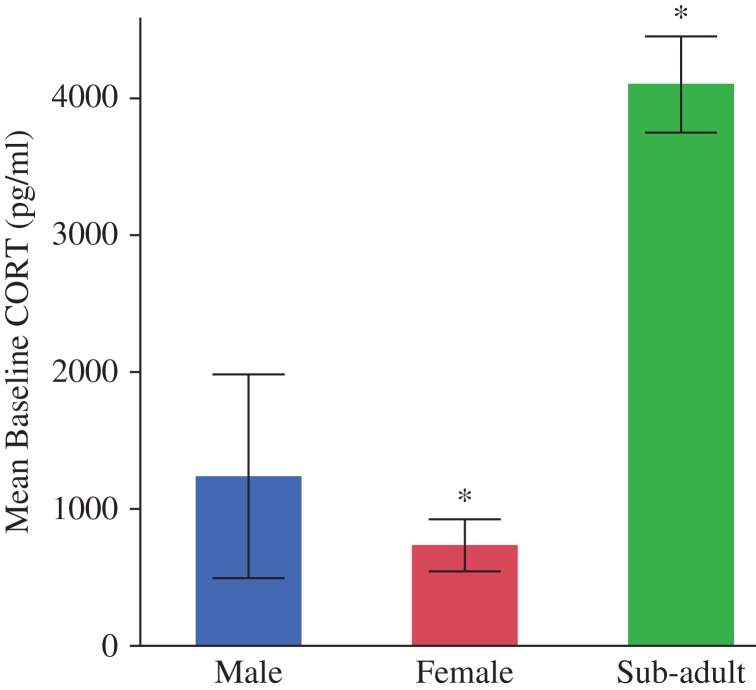
Mean (±1 SEM) baseline plasma corticosterone (CORT) concentrations in 17 wild, free-ranging *Astrochelys radiata* from southern Madagascar, February 2012. Bars with asterisks are significantly different from each other.

**Figure 3:**
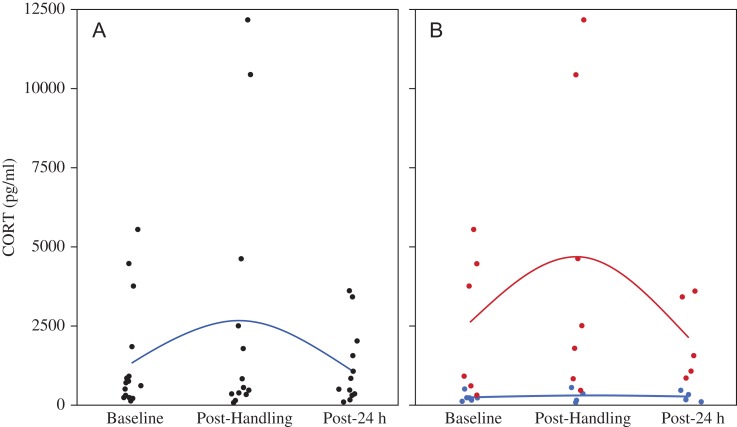
Plasma corticosterone (CORT) concentrations with mean trend line during the three sample periods for all animals combined (**A**) and separated for animals with a ‘strong’ stress response (≥75% quartile; red line) and for those with a ‘mild’ stress response (≤25% quartile; blue line) (**B**).

**Figure 4:**
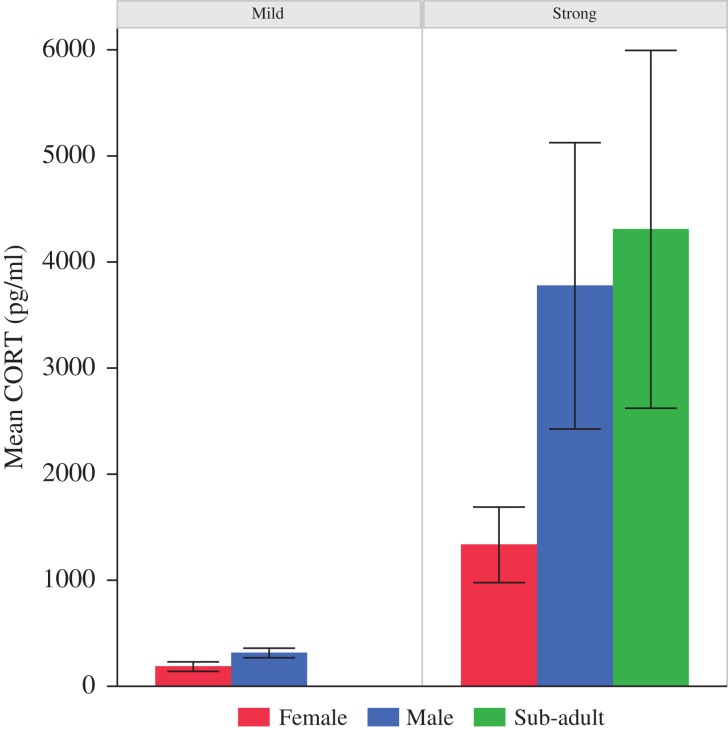
Combined average (mean ±1 SEM) corticosterone (CORT) concentration values (baseline, post-handling, and post-24 h) separated by sex for the six individuals (four males and two females) with a mild stress response and the seven individuals (three males, two females, and two sub-adults) with a strong stress response measured in a population of *Astrochelys radiata* in southern Madagascar, February 2012.

Corticosterone EIAs yielded plasma CORT concentrations ranging from 55 to 12 159 pg/ml (mean = 1657 pg/ml, median = 650 pg/ml; Fig. 1). Baseline CORT concentrations (mean = 1341 pg/ml, median = 694 pg/ml, range = 116–5538 pg/ml) were significantly different between the adult females and the sub-adults, where sub-adults averaged the highest baseline concentrations of the groups (mean = 4108 pg/ml, *F*_2,13_ = 4.745, *P* = 0.0284; Fig. 2). We were able to test for the stress response (e.g. change in CORT concentrations from baseline to post-handling values) in 12 (six males, four females, and two subadults) of the 17 animals sampled. We found no significant difference in the change of CORT concentrations attributable to handling (*F*_1,15_ = 3.119, *P* = 0.0984). We also found no differences that were explained by sex, body condition, or age class (Fig. 3A). However, we found a bimodal distribution in the slopes of the stress response (rate of CORT concentration change over time) as indicated by the notable difference between mean and median values at each time point. Stress response slopes were either well above the median (in the 75% quartile or above) or well below (in the 25% quartile or lower), generating two stress response types (*t* = 7.978, d.f. = 34, *P* < 0.001). Animals elicited either a ‘strong’ stress response to handling (131.2% mean change in plasma CORT concentrations from baseline) or a ‘mild’ stress response (56.9% mean CORT change; Fig. 4). Once separated into response types, both groups were significantly different from each other at baseline (*F*_1,11_ = 6.752, *P* = 0.0248) and post-handling (*F*_1,11_ = 5.068, *P* = 0.0458). Post-24 h samples could not be tested between the groups because of low sample sizes; however, neither response type exhibited a significant difference in CORT concentrations between baseline and post-24 h samples (mild: *P* = 0.5573 and strong: 0.0541; Fig. 3B). Two individuals (one male and one female) had slightly negative slopes (decrease in CORT post-handling) from the mild group, and one sub-adult had a strongly negative slope.

### Behaviour and response personalities

Of the 17 animals sampled, we radio-tracked 12 (five males, six females, and one sub-adult) for 22 months (from February 2012 to November 2013) after the sampling events. On average, trackers collected 148 GPS relocation points (range = 42–191) for each animal, totalling 1770 location and behavioural data points. Kernel home range areas used by animals averaged 61.4 ± 9.4 ha (range = 3.4–202.5 ha). Biennial air temperatures averaged 27.9 ± 0.1°C (range = 7.1–57.1), preferred tortoise temperature and humidity averaged 28.8 ± 0.1°C (range = 13.3–51.5) and 57.5 ± 0.3% (range = 6.9–99.4), and selected vegetation cover averaged 39.4 ± 0.4% (range = 0–90).

**Figure 5:**
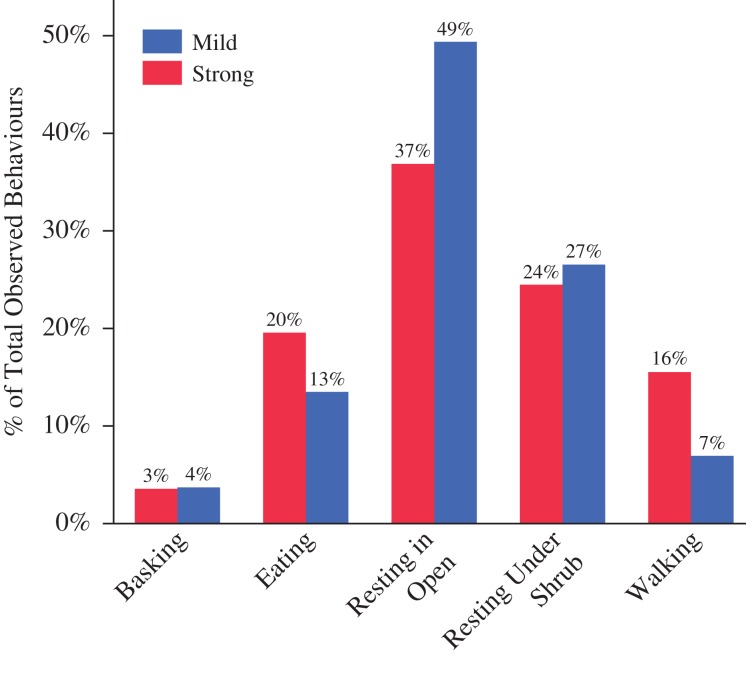
Percentage of all observed behaviours within each stress response group of wild, free-ranging *Astrochelys radiata* in southern Madagascar, between February 2012 and November 2014. *Post hoc* analyses revealed statistical differences between the response types for eating, resting in the open, and walking behaviours (*P* < 0.001).

Trackers collected those movement and activity data for nearly 2 years on nine of the individuals for which we also had stress response type data. For those, we tested their home range data, morphometric data, and CORT data separated by the two stress response types (mild vs. strong) and again found differences distinguishing the two groups. Animals exhibiting a mild stress response made longer distance movements between relocations (*F*_1,7_ = 5.85, *P* = 0.0459), maintained smaller kernel home ranges (*F*_1,5_ = 11.57, *P* = 0.0214) and had higher body condition scores (*F*_1,10_ = 7.85, *P* = 0.0188) than those exhibiting a strong stress response (Table 1). We also found that there were significant differences in the behaviour activities that the two stress response types would engage in most frequently (χ^2^ = 38.9, d.f. = 4, *P* < 0.001). Animals with a mild stress response would more often be found walking (16% of total time) or eating (20%), whereas animals with a strong stress response would more often be found resting in the open (49%) over the course of the 2 years of tracking (Fig. 5).

**Table 1:** Summary of variables assessed by stress response types (‘mild’ vs. ‘strong’ identified from corticosterone (CORT) concentration in wild, free-ranging *Astrochelys radiata* in Southern Madagascar

Significant explanatory variable	Mild (minimum–maximum) (*n* = 6)	Strong (minimum–maximum) (*n* = 6)	*P*-value
CORT (pg/ml)	234 (116–500)	2469 (314–5538)	0.0002
CORT slope (pg/ml/min)	5.3 (−32.0–27.4)	219.3 (−119.2–524.4)	0.0010
Size (SCL; mm)	331 (310–353)	262 (158–347)	0.0147
Weight (kg)	7.1 (5.5–8.2)	4.3 (0.8–7.8)	0.0072
BCS (kg/mm)	21.5 (17.7–23.6)	14.9 (4.7–22.5)	0.0188
Home range size (ha)	37.8 (25.6–49.6)	85.0 (3.4–202.5)	0.0214
Steplength (m)	37.6 (1.0–991.3)	14.7 (1.0–1228.0)	0.0459
Behaviour	↑ Walking and ↑ eating	↑ Resting in the open	<0.001

Abbreviations: BCS, body condition score; SCL, straight carapace length; and steplength, Euclidian distance between tracking locations.

## Discussion

### Corticosterone concentrations

Here, we present the first reported plasma CORT range measured in wild, free-ranging *A. radiata*. The baseline values herein can be used as a benchmark in assessing the general health of other populations or improving captive conditions of *A. radiata*. Surprisingly, we found that the CORT concentrations were widely variable within this natural population (>200-fold differences after handling), as well as finding significant variability in the baseline data (nearly a 50-fold range). Some variation is expected between age classes, because CORT has been shown to decrease with age in several taxa (Fourie and Bernstein 2011; Fourie *et al*., 2016; Mateo, 2006). This juvenile-associated high is generally attributed to mobilization of glucose for facilitation of growth and energy storage, as well as to rapidly promote memory, learning, and survival behaviours (e.g. anti-predator responses and foraging strategies). Although the baseline CORT values of our population were comparable to some other tortoise studies (Lance *et al*., 2001), the values we measured were generally much lower than those that have been measured in other tortoise species. For example, in *Gopherus agassizii* (desert tortoises), baseline CORT concentrations during the peak active season measured 7.6 ng/ml, but did not vary as widely even when stress was induced by handling or by ACTH injection (maximal <5-fold change; Drake *et al*., 2012). Similar to *G. agassizii*, sister species *G. polyphemus* studied in Georgia, USA, averaged 7.1 ng/ml CORT, but decreased to 4.9 ng/ml after 8 h of capture and manipulation in the laboratory, before returning to baseline levels 4 weeks later after having been returned to the wild (Kahn *et al*., 2007). Likewise, in our study three individuals had a negative slope (decreased CORT concentrations) after handling. We might expect a decrease in circulating CORT concentrations hours after imposing a stressor as a result of an overcompensation of the negative feedback loop CORT imposes on the hypothalamic–pituitary–adrenal axis. However, this relatively rapid decrease is likely to reflect metabolic clearance of circulating CORT in conditions of no CORT response to handling.

In an overall average within our sample, we found there to be no difference in CORT attributable to handling. Although a clear stress response has been shown after 90 min of capture and transport in *Testudo hermanni* (Hermann's tortoise; Fazio *et al*., 2014), at both 30 and 60 min in *Lepidochelys kempii* (Kemp's ridley sea turtle; Gregory and Schmid, 2001), and even as far into capture as 1–5 h for *Eretmochelys imbricata* (hawksbill turtle; Jessop *et al*., 2004), our capture window could have been too short to detect whether CORT concentrations continued to increase after post-processing release. Conversely, another study on *G. polyphemus* in southern USA concluded that trapping and handling tortoises did not affect mean CORT concentrations until after 12 h (Ott *et al*., 2000), and we did not sample during that period. There is a chance that CORT concentrations would have continued generally to increase after 12 h, as in *G. polyphemus*, but would have to return to near-baseline concentrations by the post-24 h measurement. However, it is unknown exactly how long the animal had been entrapped within the Ott *et al*. (2000) 2 h trapping window before initial sampling. Researchers sampled at the 12 h mark in a subset of the study animals, and did not sample between the initial sample and 12 h later. Therefore, it is unknown whether the animals had already elicited a stress response while inside the trap, causing ‘baseline’ samples to be skewed high, or if the animals elicited a stronger response to the trapping and handling between the two sampling periods. Additionally, the use of mean values in that study might have clouded any individual variation or response types that could have been present (Cockrem, 2013).

We argue that the broad range in CORT responses evident might represent phenotypic plasticity that is key to survival. Thus, the use of mean values, which did not reflect the bimodal response well, might be the reason why so few other chelonian studies report similar behavioural–physiological links. However, Henen *et al*. (1998) and Bradshaw (2003) suggest that extreme variability may be indicative of one the following three strategies in tortoises; (i) heterostasis, where the animals have high variability as an adaptive strategy; (ii) anhomeostasis, where the animal relinquishes a more stable homeostasis only temporarily in order to survive current conditions, and we happen to capture the animal at one of these times (see also ‘emergency life-history stage’; Wingfield *et al*., 1998); or (iii) the animal is currently extremely stressed in its habitat and struggling for survival. This last option implies that chronic stress may have deleterious implications on overall demography and population health.

### Behaviour and response personalities

We collected movement and activity data for nearly 2 years on 12 individuals in the present study, which we used to calculate home ranges and quantify activity. Of those 12, nine were also in the group for which we could assign a stress response type (mild vs. strong). Although our sample sizes are somewhat small, they are comparable to other recent endocrine studies on wild chelonians (Kahn *et al*., 2007; Baxter-Gilbert *et al*., 2014; Fazio *et al*., 2014; Hunt *et al*., 2016; Winters *et al*., 2016). Within our sample data set, we detected two distinct stress response types correlated to a small suite of behaviours and traits among a population of wild, free-ranging *A. radiata*, which we propose may represent distinct personalities.

There are generally five animal personality categories: shyness–boldness, exploration–avoidance, activity, sociability and aggressiveness (Sih *et al*., 2004; Réale *et al*., 2007). The five categories of personalities have recently been grouped and spread over a spectrum of two extremes, proactive and reactive. The proactive–reactive axis comprised a spectrum of behaviours from aggressive and bold individuals exhibiting exploratory, consistent and environmentally manipulative tendencies at one end, contrasted with careful, shy and sensitive to change (reactive) at the other end (Koolhaas *et al*., 1999; Sih *et al*., 2004). Aggression and boldness have been consistently linked in lockstep in response to certain stimuli, such as presence of predators (Bell and Sih, 2007). Proactive individuals are aggressive and mediate stressors, generally correlated with the sympathetic nervous system, whereas higher glucocorticoid concentrations associated with hypothalamic–pituitary–adrenal axis stimulation generally correlate with passive or reactive individuals (Koolhaas, 2008). As has been shown in birds (Cockrem, 2007), we also were able to identify animals in the bold, proactive category, with larger body sizes and better body condition. The animals maintained smaller home ranges but moved more within those ranges while walking and eating, and showed only a mild stress response to handling. We conversely identified individuals that could be classified as reactive, with smaller body sizes and lower body condition scores, larger home ranges but moving less, and often found resting, and showing strong changes in CORT concentrations. These consistent differences in behavioural traits among the CORT response types are further evidence for the group distinctions (animal personality) in this species. Few other studies have investigated chelonian physiological–behavioural traits, but there have been a few that may be considered.

Ruby *et al*. (1994) documented that immature *G. agassizii* would move further than other groups when water stressed. Overall, they found no differences based on sex or stressed group on feeding, or movement activity, but the researchers did see overall differences by month and in one of the years. During the first year of the study, tortoises that were water stressed moved more frequently, maintained larger home ranges and were more often found in novel locations than non-stressed animals. Ruby *et al*. (1994) also found that feeding and movement activity were interrelated, exactly as we found in the present study.

Another study in hatchling *Trachemys scripta* (red-eared slider turtles) found robust and stable personalities (Carter *et al*., 2016). However, many studies appear to stop short of making a behavioural–physiological connection. In a study involving *G. polyphemus*, authors were generally unable to correlate home range sizes, number of burrows used, activity, or body size with plasma CORT, and therefore, were not able to identify personalities within their study population (Ott *et al*., 2000). However, that investigation did find correlations within the sexes involving body sizes and number of burrows used, suggesting that with more detailed sampling and monitoring, groupings of traits might become more clear. Likewise, in a study on a population of *Graptemys flavimaculata* (yellow-blotched sawback turtles) in Mississippi, Selman *et al*. (2012) found that the CORT concentrations of capture-stressed animals varied significantly over the year and suggested correlations with natural history traits, but did not further investigate personality traits. Finally, in a 2-month study that involved handling manipulation and subsequent radiotelemetry monitoring of 10 *G. polyphemus*, tortoises moved more frequently in the 4 weeks after manipulation compared with the 4 weeks before, yet the five control tortoises showed no differences in movements (Kahn *et al*., 2007). These differences were in spite of the fact that Kahn *et al*. (2007) were not able to detect significant differences in CORT during their sampling; however, in that study, tortoises were not sampled until after 8 h of captivity, and there was a drop in CORT by that time. Lack of significance in associating behaviours and stress response could be attributable to the relatively small sample size or extreme duration between sampling events, yet the authors concluded that their extensive handling procedures had no effect on the study animals and made comparisons with literature on captive animals. With careful sampling procedures and improved attention to monitoring of behaviours for longer periods of time, literature on chelonian personalities in response to a variety of factors is likely to emerge with increased frequency in the coming years.

Many of the aforementioned studies used mean values to compare control and treatment groups, with little or no mention of investigations into variations within those groups. If we had followed suit and not investigated the CORT variation we detected, we likewise would not have detected any significant differences in CORT attributable to handling or associated with behavioural traits or physical condition within this population. The power of the golden mean is still strong and deeply ingrained in wildlife research studies.

The combination of personality traits provides a window into the evolution of the life-history traits of the species, as particular combinations and variability may be selectively adaptive (Dall *et al*., 2004; Biro and Stamps, 2008; Lapiedra *et al*., 2017). In our study, we found that animals maintaining larger home ranges were less likely to be active frequently and exhibited a strong stress response, suggesting that this group might be both explorative and display high shyness in response to risky situations, but were also those individuals which had smaller body sizes and lower body condition scores. These individuals might be undergoing ontogenetic growth, searching for forage or uninhabited home ranges, or recovering from injury or conflict. All of these situations would be likely to cause the individual to have higher baseline CORT and a hair-trigger stress response to aid the animal in finding refugia in which to grow and/or recover, whereas those individuals with larger body sizes and better body conditions need not maintain large home ranges, have the reserves to be more active and forage frequently, and consequently, are bolder, with only mild stress responses. Variability in personality traits allows the individual to acclimate to stochastic conditions and survive to reproduce, maintaining the variability in the population.
